# Supplementary material for: Medically Tailored Grocery Deliveries to Improve Food Security and Hypertension in Underserved Groups: A Student-Run Pilot Randomized Controlled Trial
Source: Healthcare (Basel). 2025 Jan 27;13(3):253. doi: 10.3390/healthcare13030253 (PMC11817985; doi:10.3390/healthcare13030253)
Supplement: Supplementary file 1 [file healthcare-13-00253-s001.zip › healthcare-3373078-supplementary/Supplementary_1.27.25/Table S1_.pdf]

Cluster	Code	Definition	Exemplar
	**Category: Food Provisions**		
Satisfaction	Taste	Enjoyment associated with the flavor of included food items	"muy rico la comida y saludable" *[delicious food and healthy]*<br><br>"The classes were helpful, giving me to think about how nutrition is so important, and how nutrition does NOT mean bland!! :)"
	Variety	Inclusion of diverse types of produce and other food items	"Learned to cook differently. I thought of the variety of produce as a TV show 'Chopped' made food tasty"<br><br>"Fruits and veggies of all kind were very much appreciated."
	Freshness	Perception that food has been recently harvested or has not decayed	"Like the delivery of fresh foods."<br><br>"Produce fresh and delicious."
	Satisfaction	Fulfillment of expectations engendered by food item(s)	"All in all the food is good and delicious"<br><br>"The food was very good."
	Alignment with preferences	Concordance between a food item or traits of a food item and one's cultural, social, dietary or other predilections	"muy bien"[†] *[very good]*<br><br>"Very Good"[†]
	Healthiness	Perception that food has high nutritional value	"muy bien aprendi mucho sobre la comida y muy saludable"*[Very good. Leaned a lot about food and very healthy.]*<br><br>"muy rico la comida y saludable" *[delicious food and healthy]*
	**Knowledge**		
	Practical / cooking skills	Reported they had learned new food preparation skills, especially for healthy foods	"I like the recipes, giving me better idea of how to prepare different dishes."<br><br>"Fueron muy importantes aprendi mucho sobre como cosinar saludable y que la comida estubiera rico." *[They were very important, I learned a lot about how to cook healthy and make the food delicious.]*
	Recipes	Satisfaction with set of instructions for preparing a particular dish, including a list of the ingredients and tools required	"loved the recipes"<br><br>"Very satisfied with staff (patient, kind, helpfulness). Well trained/Learning staff. 1. I really enjoyed presentations of food/recipes. 2. Produce fresh and delicious. 3. Learned new ways to cook and prepare food."
	Disease management	Understanding of tangible dietary patterns and habits one may develop to control or improve their health condition	"The knowledge given being a diabetic was helpful & I changed my habits"<br><br>"Thank yall & appreciate y'all this was important. I got my AIC down 3 points!"

	Teaching / classes	NCCU dietetic interns provided impactful instructional sessions as part of the study intervention	"Very Goodñ Taught very well"<br><br>"Well presented. Informative"
	Food / nutrition knowledge	Improved understanding of what foods promoted health	"muy bien aprendi mucho sobre la comida y muy saludable" *[very rich the food and very healthy]*<br><br>"muy bueno el programa aprendi mucho / La verdad todo el progra estubo muy bueno" *[Very good. Leaned a lot about food and very healthy.]*
	**Supports**		
	Food access	The stable availability of nourishing, affordable, and suitable foods	"It helped in terms of the groceries."<br><br>"Really good experiment, very helpful for a senior like myself to have fresh produce"
	Financial relief	Direct support circumventing cost-associated barriers to consuming fresh foods	"I feel it is a Big help for the community you don´t always have money to buy fruit and veggies"[*]
	Home visits	Intervention in which study team personnel meet with a participant in their place of living for the purpose of conducting study activities	"She wanted to share that everyone who came out to the house was very informative, and she appreciated being well-informed about what was going on."[*]
	Community benefit	Perception that intervention would positively affect the lived environment of oneself or one's neighbors	"I feel it is a Big help for the community you don´t always have money to buy fruit and veggies"<br><br>"This is a great program and should continue in order to benefit more residents in need."
	Delivery	Food items being brought directly to the home of the study participant was a value add for the program	"Like the delivery of fresh foods."[*]
	**Agency**		
	Relationship-building	Facilitation of building mutual familiarity or understanding among or between individuals through the intervention	"The program was excellent. It was very enjoyable. I got to meet different types of people as well."[*]

	Knowledge-sharing	Pariticipant reported disseminating information learned within their social network	"She was able to pass along what she learned to her children."[*]
	Experimentation	Expanded opportunities to incorporate new foods and diversify diets	"Different in some ways. But allowed myself to experiment with some choices."<br><br>"Experimented with spaghetti squash and enjoyed it. "
	**Study logistics**		
	Information sharing	Adequacy of methods used by study personnel to communicate details about the study to study participants	"Well presented. Informative"<br><br>"She wanted to share that everyone who came out to the house was very informative, and she appreciated being well-informed about what was going on. "
	Staff	Study participants' appreciation of study personnel helpfulness	"Thank yall & appreciate y'all this was important. I got my AIC down 3 points!"<br><br>"Very satisfied with staff (patient, kind, helpfulness). Well trained/Learning staff."
	**General Endorsement**		
	Summative Evaluation	At the end of the study, participants expressed global satisfaction with the program overall	"Thank yall & appreciate y'all this was important. I got my AIC down 3 points!"<br><br>"muy bueno el programa aprendi mucho / La verdad todo el progra estubo muy bueno" *[The program was very good I learned a lot / The truth is the whole program was very good.]*
Dissatisfaction	**Food Provisions**		
	Lack of freshness	State of decay deemed unappetizing or not suitable for human consumption	"A very few of the produce wasn't so fresh (corn)"[*]
	Unfamiliarity	Lack of prior experience or knowledge about a food item and/or its integration into food preparation	"Some of the food I did not know how to prepare (eggplant)"<br><br>"Didn't know what to do with squash."
	Misalignment with preferences	Discordance between a food item or traits of a food item and one's cultural, social, dietary or other predilections	"About 80%, and it made them try squash for the first time."<br><br>"She said it matched around 60%. She doesn't like raspberries but preferred the strawberries."

* Limited exemplars due to small sample size

† Responses correspond to Question 2 in Figure 1
