# Supplementary figures and images for: Medically Tailored Grocery Deliveries to Improve Food Security and Hypertension in Underserved Groups: A Student-Run Pilot Randomized Controlled Trial
Source: Healthcare (Basel). 2025 Jan 27;13(3):253. doi: 10.3390/healthcare13030253 (PMC11817985; doi:10.3390/healthcare13030253)

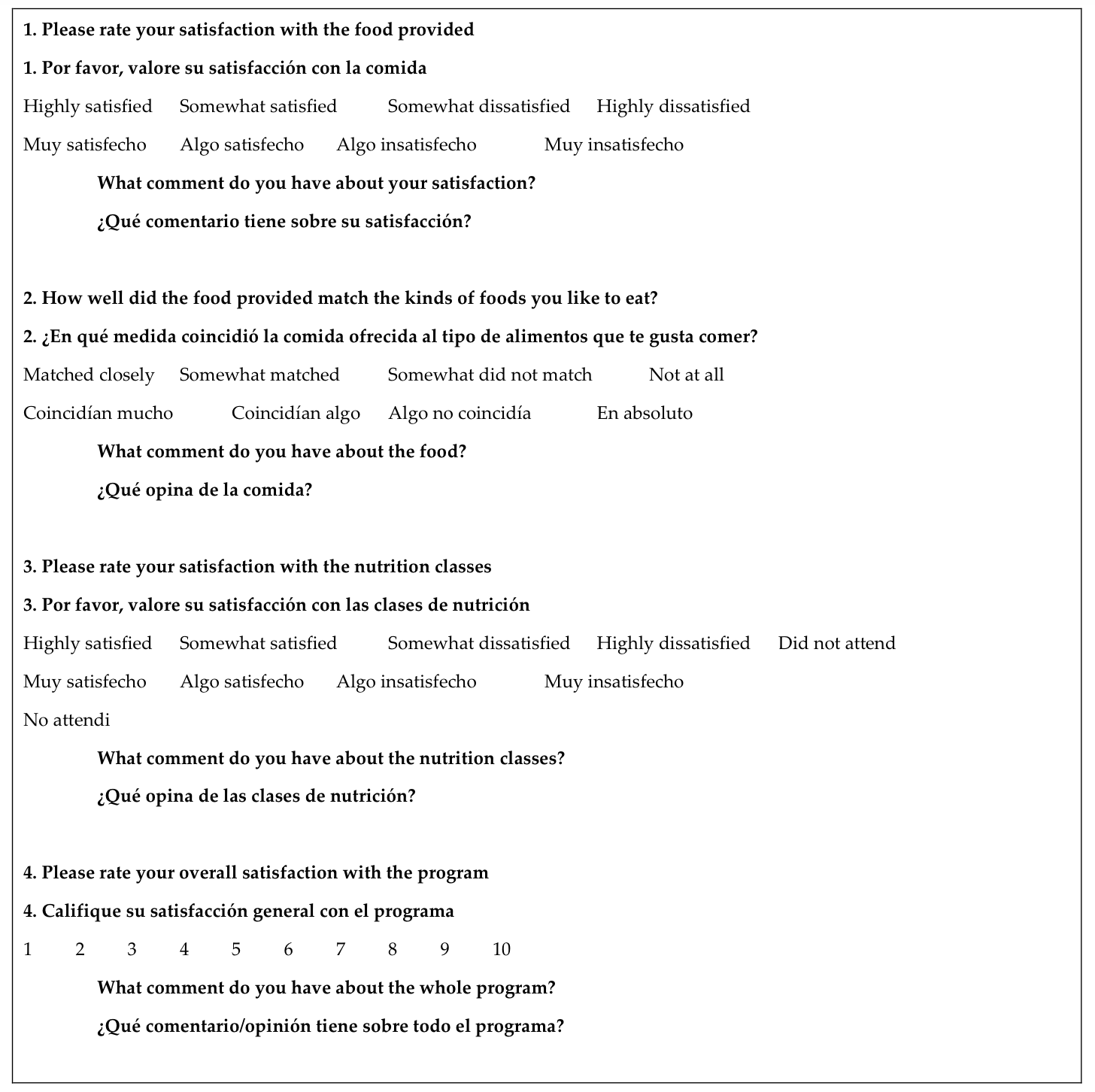

Supplement: Supplementary file 1 [file healthcare-13-00253-s001.zip › healthcare-3373078-supplementary/Supplementary_1.27.25/Figure S1.png]

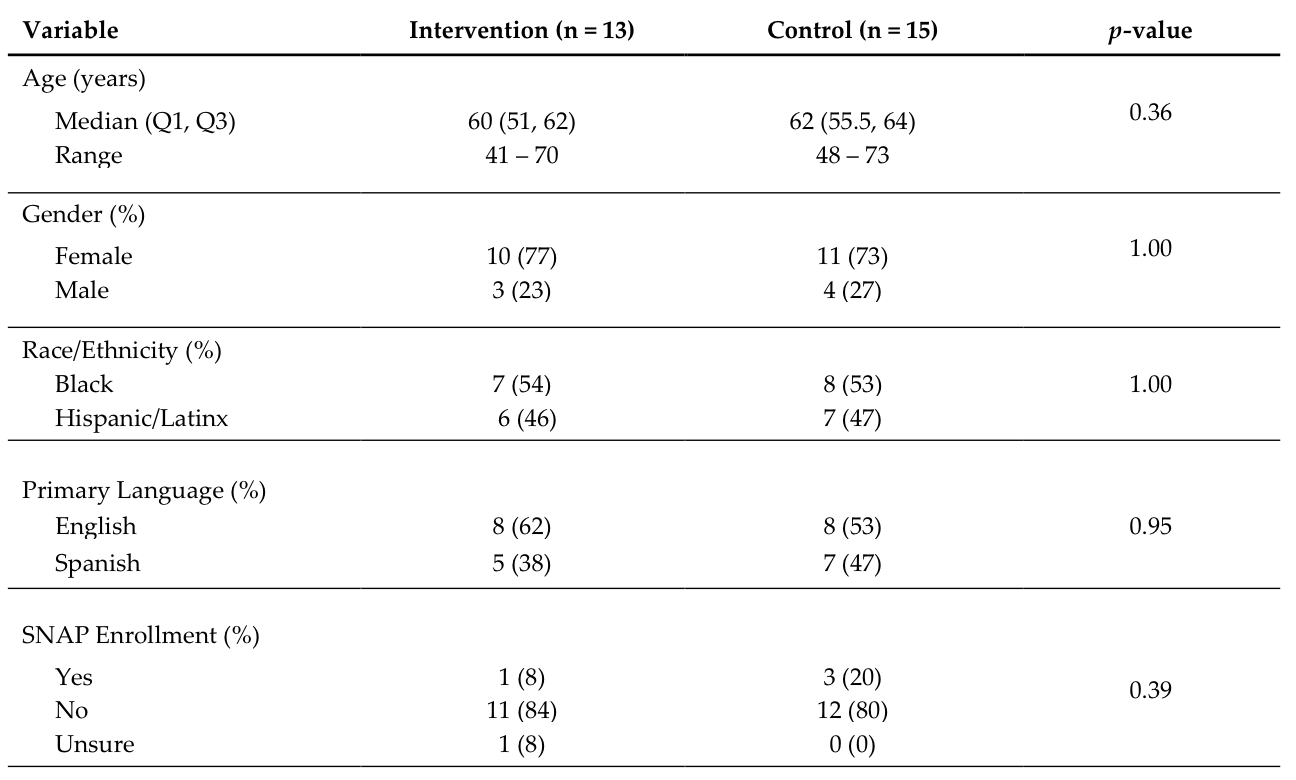

Supplement: Supplementary file 1 [file healthcare-13-00253-s001.zip › healthcare-3373078-supplementary/Supplementary_1.27.25/Table S2.png]

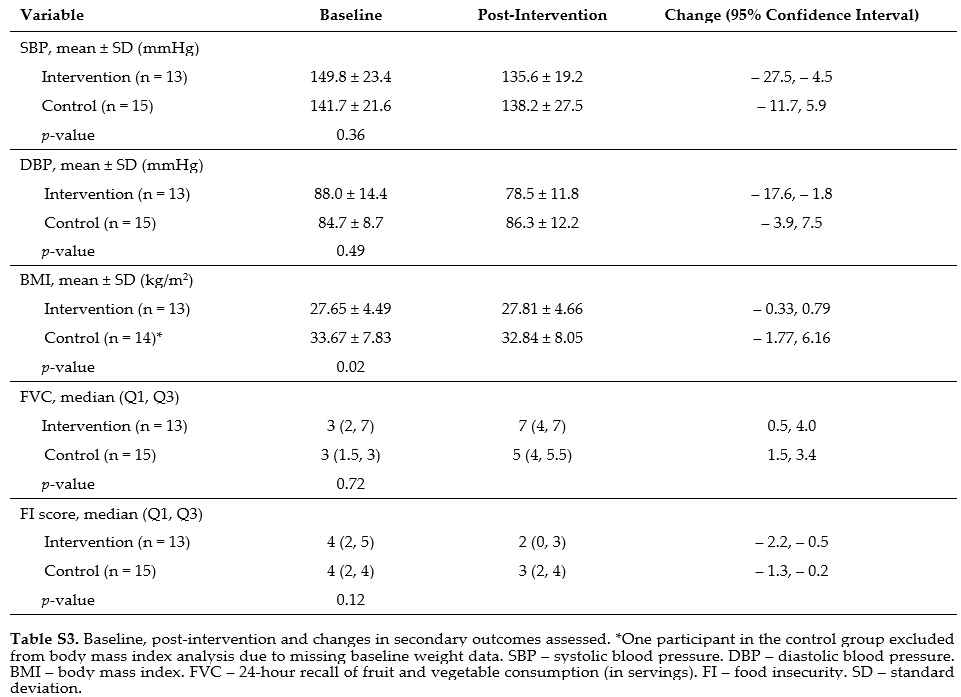

Supplement: Supplementary file 1 [file healthcare-13-00253-s001.zip › healthcare-3373078-supplementary/Supplementary_1.27.25/Table S3_.png]
